# Supplementary material for: Deep Neural Networks to Register and Annotate Cells in Moving and Deforming Nervous Systems
Source: bioRxiv. 2025 Jun 21:2024.07.18.601886. Preprint. [Version 2] doi: 10.1101/2024.07.18.601886 (PMC12262252; doi:10.1101/2024.07.18.601886)
Supplement: Supplement 1 [file NIHPP2024.07.18.601886v2-supplement-1.pdf]

bioRxiv preprint doi: https://doi.org/10.1101/2024.07.18.601886; this version posted June 21, 2025. The copyright holder for this preprint (which was not certified by peer review) is the author/funder, who has granted bioRxiv a license to display the preprint in perpetuity. It is made available under aCC-BY 4.0 International license.

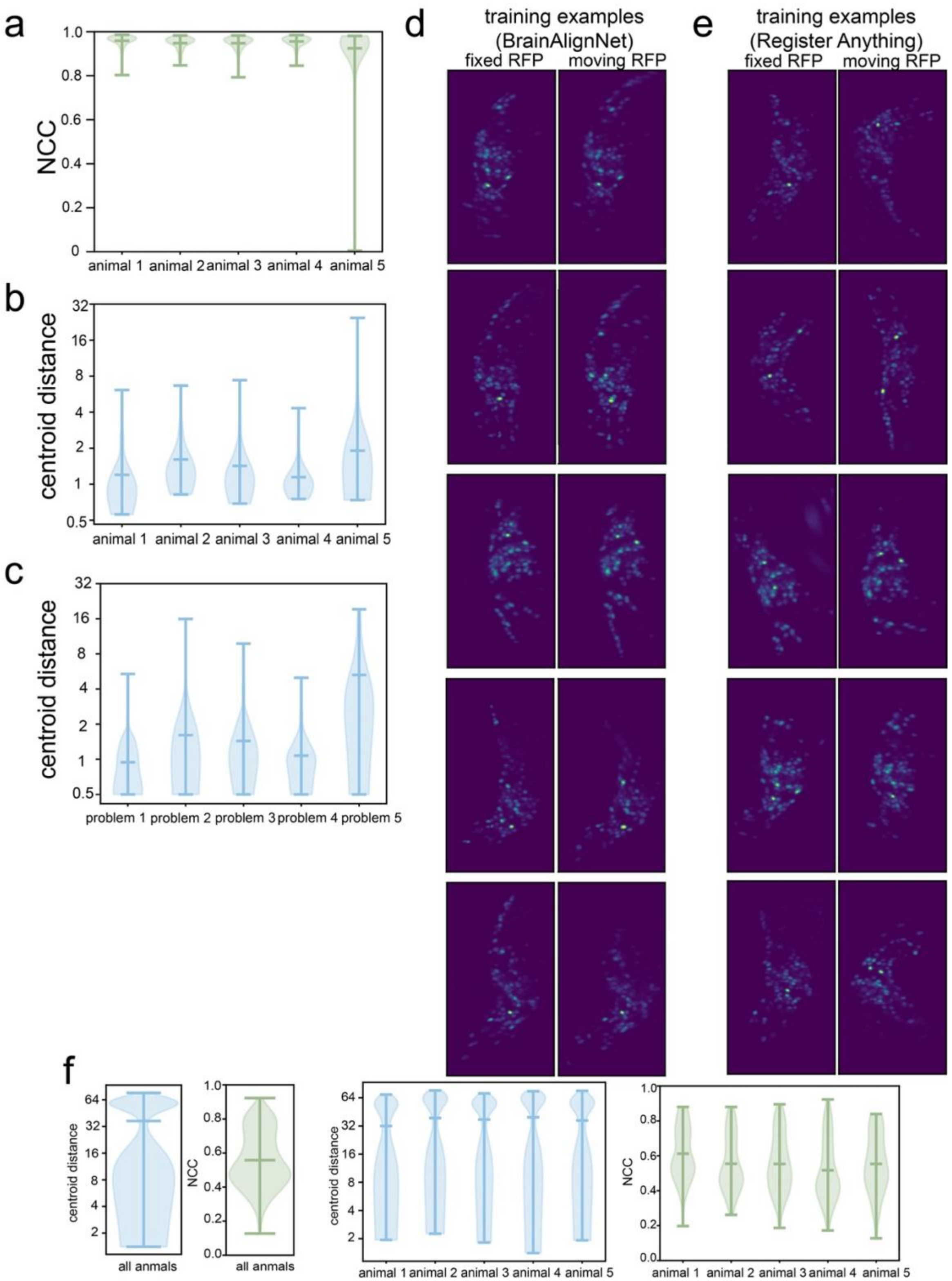

Figure S1. Example images and performance of network trained to register arbitrary image pairs.

(A) Performance of image registration in five different animals in the testing set. Normalized Cross-Correlation (NCC) scores of aligned tagRFP images are shown, which indicate the

bioRxiv preprint doi: https://doi.org/10.1101/2024.07.18.601886; this version posted June 21, 2025. The copyright holder for this preprint (which was not certified by peer review) is the author/funder, who has granted bioRxiv a license to display the preprint in perpetuity. It is made available under aCC-BY 4.0 International license.

extent of image alignment (best achievable score is 1). 90-100 registration problems examined per animal are shown as violin plots with the overlaying lines indicating minimum, mean, and maximum values.

(B) Performance of image registration in five different animals in the testing set. Centroid distance is the average Euclidean distance between the centroids of matched neurons in each image (best achievable score is 0). 90-100 registration problems examined per animal are shown as violin plots with the overlaying lines indicating minimum, mean, and maximum values.

(C) Performance of image registration in five different registration problems (i.e. image pairs) from one example animal. Centroid distance is the average Euclidean distance between the centroids of matched neurons in that image pair (best achievable score is 0). All the centroid position distances for each registration problem as shown as violin plots with the overlaying lines indicating minimum, mean, and maximum values.

(D) Five example image pairs in the training set for BrainAlignNet. These are maximum intensity projections of the tagRFP channel, showing two different timepoints that were selected to be the fixed and moving images in each of these five registration problems.

(E) Five example image pairs in the training set for the network trained to align arbitrary image pairs, including much more challenging problems. Note that the head bending is more dissimilar for these image pairs, as compared to those in (D). Data are shown as in (D).

(F) Performance of the network trained to register arbitrary image pairs. Quantification is for testing data. We quantify centroid distance (average alignment of neuron centroids) and NCC (image similarity) as in panels (A-C). By both metrics, this network's performance is far worse than that of the BrainAlignNet presented in Fig. 1. The two panels on the right show that results are qualitatively similar for different animals in the testing set.

bioRxiv preprint doi: https://doi.org/10.1101/2024.07.18.601886; this version posted June 21, 2025. The copyright holder for this preprint (which was not certified by peer review) is the author/funder, who has granted bioRxiv a license to display the preprint in perpetuity. It is made available under aCC-BY 4.0 International license.

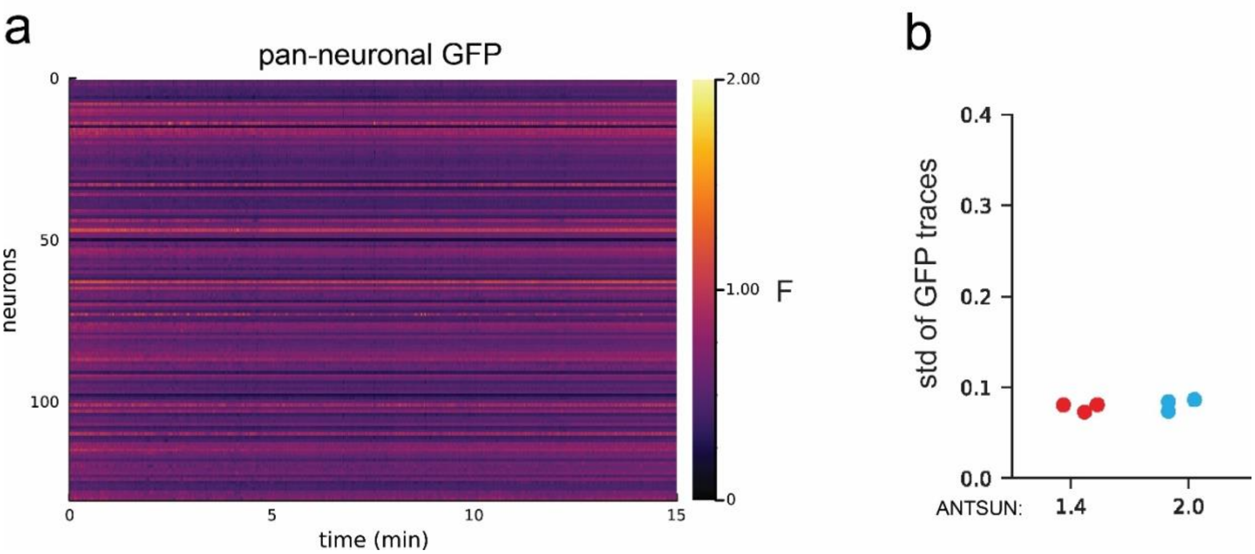

Figure S2. Characterization of pan-neuronal GFP datasets processed by ANTSUN 2.0.

(A) Example *rimb-1::GFP* (pan-neuronal GFP) dataset processed by ANTSUN 2.0. The data are shown as ratiometric GFP/RFP without any further normalization.

(B) Quantification of the standard deviation of GFP traces from 3 pan-neuronal datasets processed by either ANTSUN 1.4 (without BrainAlignNet) or 2.0 (with BrainAlignNet). To standardize across datasets, the standard deviation here was computed on traces that were normalized by F/Fmean. Ideally, GFP traces should have low standard deviation; processing with ANTSUN 2.0 did not impair trace quality, compared to the previously described ANTSUN 1.4[36].

bioRxiv preprint doi: https://doi.org/10.1101/2024.07.18.601886; this version posted June 21, 2025. The copyright holder for this preprint (which was not certified by peer review) is the author/funder, who has granted bioRxiv a license to display the preprint in perpetuity. It is made available under aCC-BY 4.0 International license.

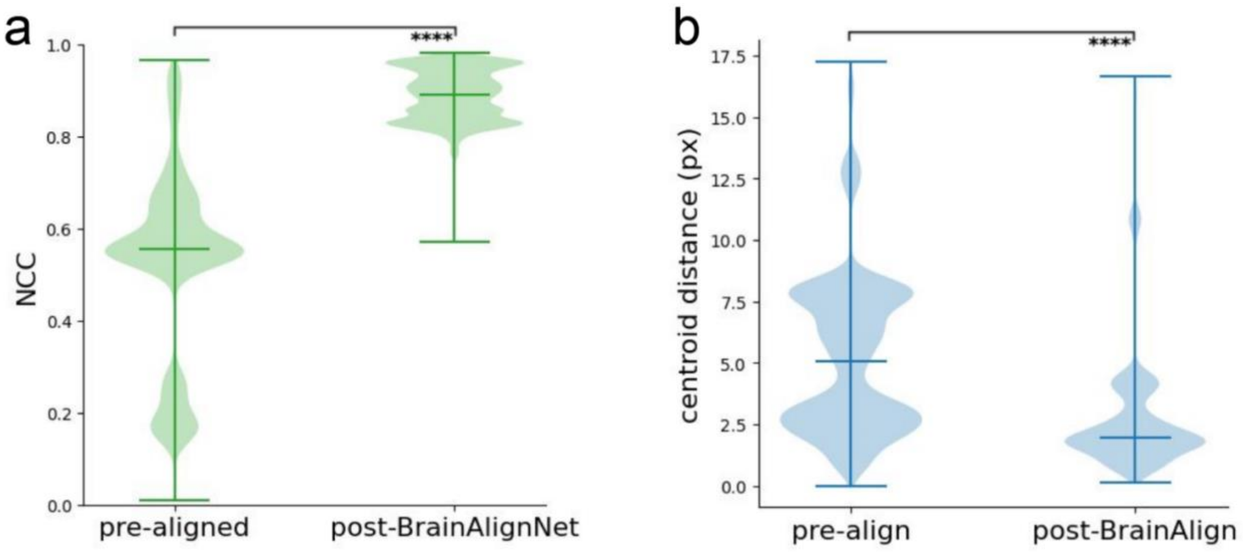

Figure S3. BrainAlignNet Performance on Additional Withheld Jellyfish Data

(A) Image registration quality was assessed via image alignment on image pairs before and after BrainAlignNet. These image pairs were from the animals used in the training data, but were different image pairs than those used for training (n = 25697). As in Fig. 3, Normalized Cross-Correlation (NCC) scores of aligned mCherry images indicate image alignment. NCC is shown between Euler initialized images (“pre-aligned”) and BrainAlignNet-registered images. ****$p<0.0001$, two-tailed Wilcoxon signed rank test.

(B) Image registration quality was assessed via centroid alignment on image pairs before and after BrainAlignNet. These image pairs were from the animals used in the training data, but were different image pairs than those used for training (n = 25697). Centroid distance is as described in Fig. 3 and is shown between Euler initialized images (“pre-align”) and BrainAlignNet-register images. ****$p<0.0001$, two-tailed Wilcoxon signed rank test.

bioRxiv preprint doi: https://doi.org/10.1101/2024.07.18.601886; this version posted June 21, 2025. The copyright holder for this preprint (which was not certified by peer review) is the author/funder, who has granted bioRxiv a license to display the preprint in perpetuity. It is made available under aCC-BY 4.0 International license.

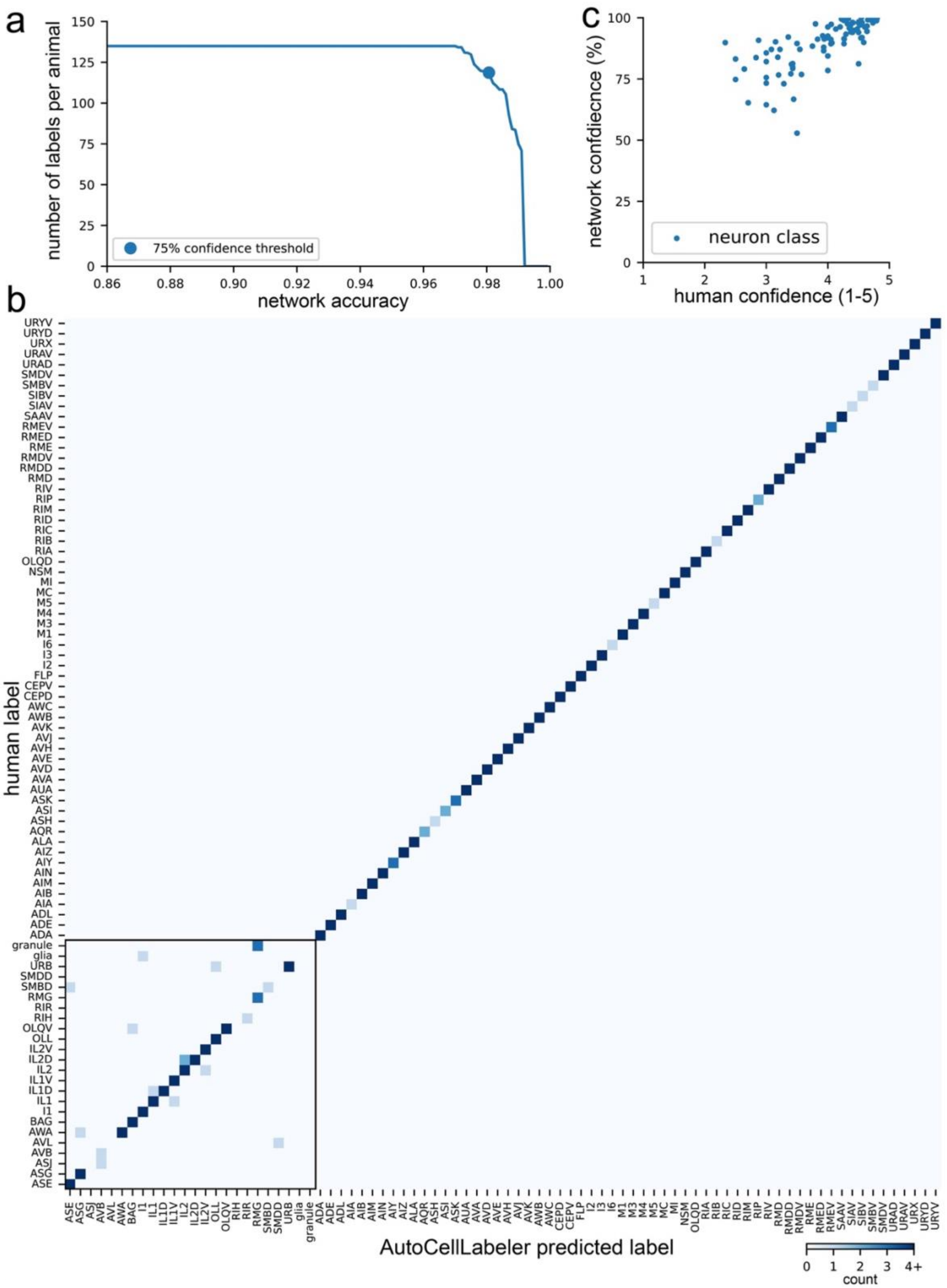

Figure S4. Further characterization of the AutoCellLabeler network

(A) Tradeoff of network labeling accuracy (x-axis) and number of neurons labeled (y-axis) for the full AutoCellLabeler network. The number of neurons labeled can be varied by

bioRxiv preprint doi: https://doi.org/10.1101/2024.07.18.601886; this version posted June 21, 2025. The copyright holder for this preprint (which was not certified by peer review) is the author/funder, who has granted bioRxiv a license to display the preprint in perpetuity. It is made available under aCC-BY 4.0 International license.

adjusting the threshold confidence that the network needs to achieve to label an ROI. By varying this threshold, we were able to generate this curve. This full curve captures the tradeoff and shows the 75% confidence threshold (blue circle) that we selected to use in our analyses.

(B) Confusion matrix showing which neurons could potentially be confused for one another by AutoCellLabeler. Note that, except for the diagonal, the matrix is mostly white, reflecting that it is mostly (98%) accurate. Neurons with some inaccuracies were clustered to the lower left (boxed region). Note that with a linear color scale the diagonal would be off-scale bright with correct labels. So we capped the colorbar range at 4 counts so as to not block the ability to see actual confusion entries. For reference, the actual mean value across the diagonal is 9.7.

(C) Positive correlation between human and autolabel confidence across the neuronal cell types (each cell type is a blue dot). This plot also highlights that a subset of cells are more difficult for human labelers and, therefore, also for AutoCellLabeler (i.e. the cells that are not clustered in the upper right).

bioRxiv preprint doi: https://doi.org/10.1101/2024.07.18.601886; this version posted June 21, 2025. The copyright holder for this preprint (which was not certified by peer review) is the author/funder, who has granted bioRxiv a license to display the preprint in perpetuity. It is made available under aCC-BY 4.0 International license.

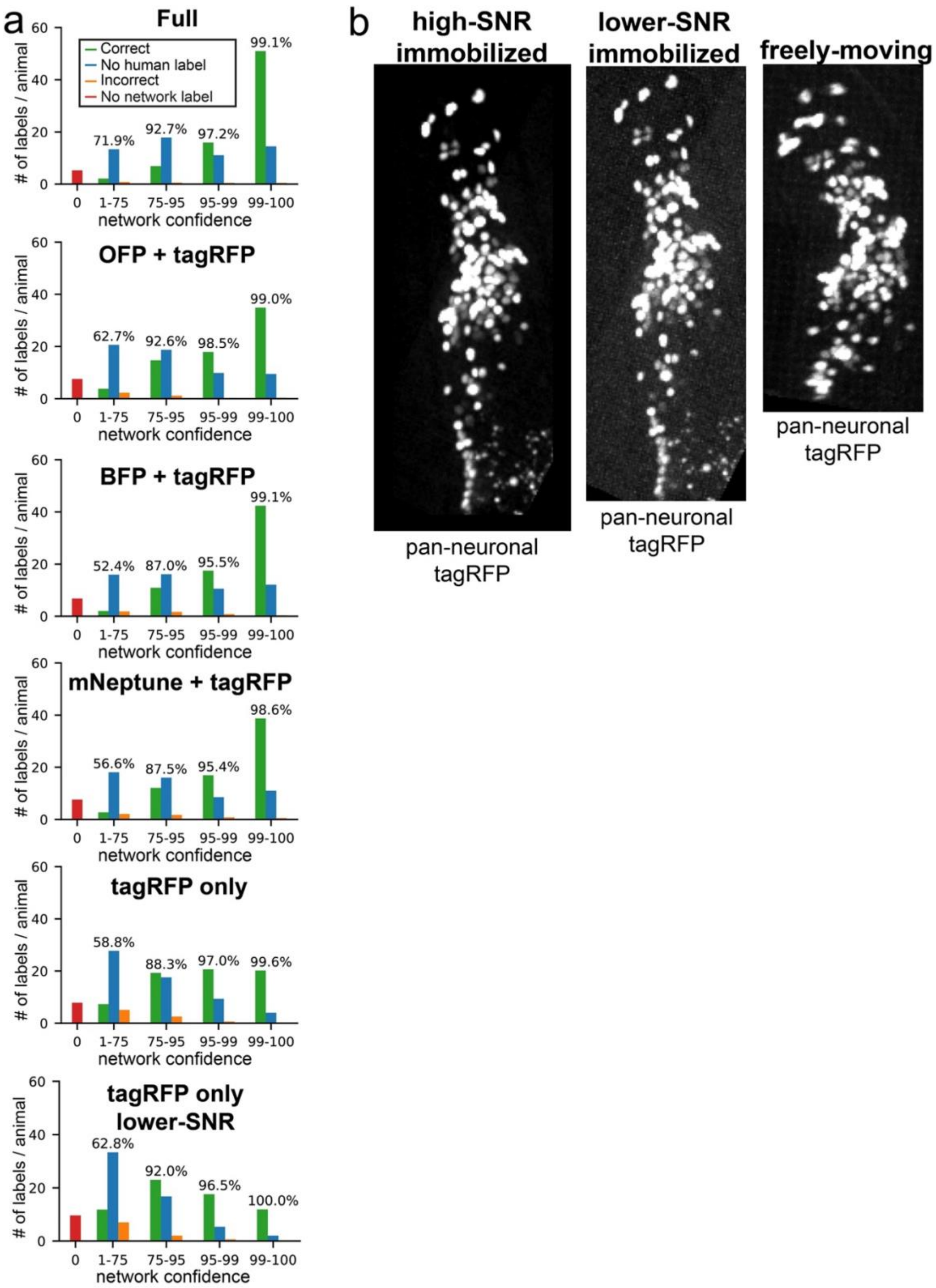

Figure S5. Further characterization of the different AutoCellLabeler variants.

(A) These plots, displayed as in Fig. 4F, show the performance of different indicated cell annotation networks (trained and/or evaluated on different fluorophores, as indicated).

bioRxiv preprint doi: https://doi.org/10.1101/2024.07.18.601886; this version posted June 21, 2025. The copyright holder for this preprint (which was not certified by peer review) is the author/funder, who has granted bioRxiv a license to display the preprint in perpetuity. It is made available under aCC-BY 4.0 International license.

Data are displayed to show network performance on different ROIs that it labels with different levels of confidence. Printed percentage values are the accuracy of AutoCellLabeler within the corresponding confidence category, computed as $\frac{correct}{correct+incorrect}$. Note that the lower performing networks (for example, tagRFP-only) are still accurate for their high-confidence labels, and that their decreased accuracy is mostly due to a lower fraction of high-confidence labels (i.e. more cell types where the networks had low confidence in their annotations).

(B) Example maximum intensity projection images of the worm in the tagRFP channel under three different imaging conditions: immobilized high-SNR (created by averaging together 60 immobilized lower-SNR images together, our typical condition for NeuroPAL imaging); immobilized lower-SNR (i.e. one of those 60 images); and freely moving (which was taken with the same imaging settings as immobilized lower-SNR but in a freely moving animal)

bioRxiv preprint doi: https://doi.org/10.1101/2024.07.18.601886; this version posted June 21, 2025. The copyright holder for this preprint (which was not certified by peer review) is the author/funder, who has granted bioRxiv a license to display the preprint in perpetuity. It is made available under aCC-BY 4.0 International license.

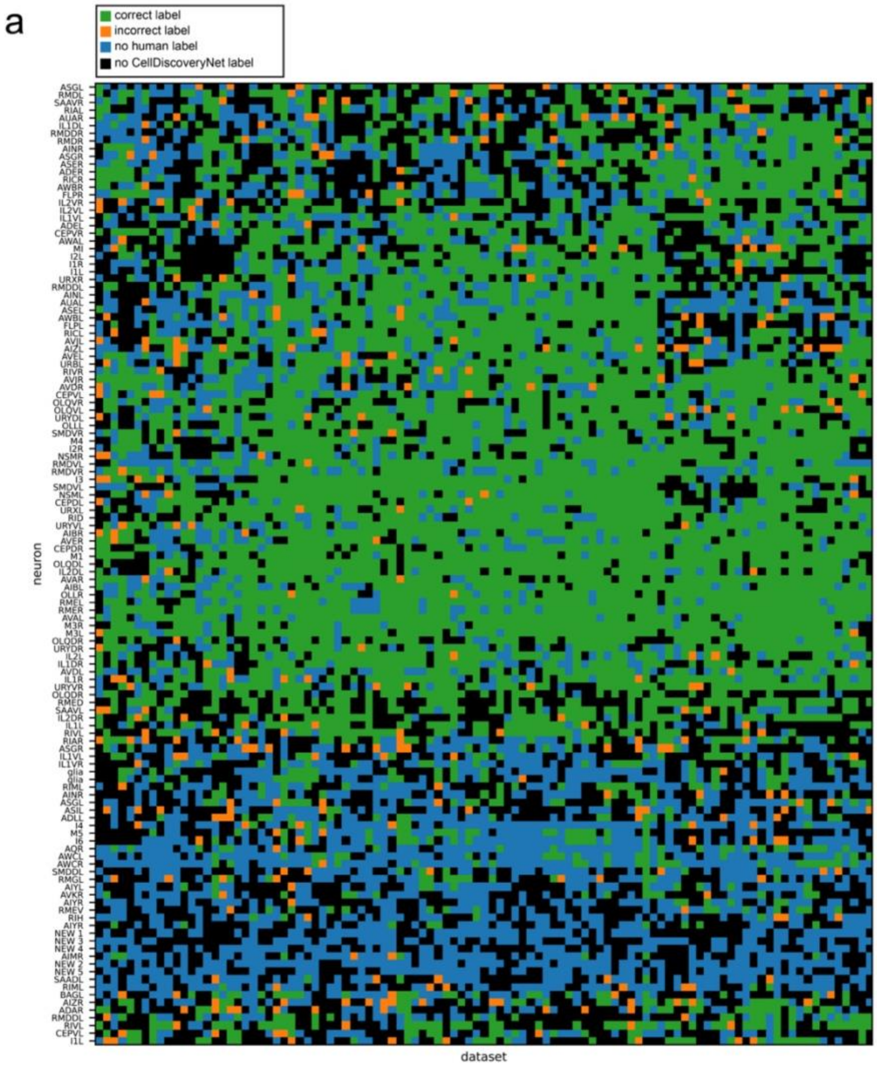

Figure S6. Further characterization of CellDiscoveryNet and ANTSUN 2U performance

(A) Matrix of all clusters generated by running ANTSUN 2U. Each row is a distinct cluster (i.e. inferred cell type), while each column is a distinct animal. Black entries mean that the given cluster did not include any ROIs in the given animal (ie: ANTSUN 2U failed to label that cluster in that animal). Non-black entries mean that the cluster contained an ROI in that animal. Row names correspond to the most frequent human label among ROIs in the cluster (this was defined by first disambiguating most frequent neuron class, and then disambiguating L from R). Green entries correspond to cases when the given ROI's label matched the most frequent class label (row name ignoring L/R), orange entries correspond to the case when the given ROI's label did not match the most frequent class label, and blue entries mean that the given ROI did not have a high-

bioRxiv preprint doi: https://doi.org/10.1101/2024.07.18.601886; this version posted June 21, 2025. The copyright holder for this preprint (which was not certified by peer review) is the author/funder, who has granted bioRxiv a license to display the preprint in perpetuity. It is made available under aCC-BY 4.0 International license.

confidence human label. The neurons “NEW 1” through “NEW 5” are clusters that were not labeled frequently enough by humans to be able to determine which neuron class they corresponded to, as described in the main text. Note that there are two rows of “glia” potentially corresponding to two different types of glia in different stereotyped locations (though in all labeling in this paper, glia are just given a single label type rather than subsetting to subtypes of glia)
